# Metatranscriptomics reveals the horse gut RNA virome and a viral sharing network with human and domestic animals

**DOI:** 10.3389/fvets.2026.1755551

**Published:** 2026-03-03

**Authors:** Can Li, Kaiping Liu, Ni Wei, Shengwei Hu, Xiaoyue Li, Cunyuan Li

**Affiliations:** College of Life Sciences, Shihezi University, Shihezi, Xinjiang, China

**Keywords:** horse, horse gut virome, metatranscriptome, RNA virus, sharing network

## Abstract

**Introduction:**

RNA viruses, a unique class of life forms, are widely distributed in nature and pose potential health risks. Monitoring the gut RNA virome in livestock is a crucial component of global health surveillance. As important companion animals, horses play a vital role in transportation and make significant contributions to various cultural and economic activities. Nevertheless, the characteristics of horse gut RNA viruses remain largely uncharted.

**Methods:**

In this study, we used metatranscriptome sequencing and bioinformatics methods to characterize viruses within the gut contents of 16 horses spanning three breeds (Thoroughbred, Akhal-Teke and Yili horse).

**Results:**

A total of 497 viral genomes from 22 viral families were recovered, including both double-stranded RNA (dsRNA) and single-stranded RNA (ssRNA) viruses, and encompassed positive- and negative-sense types commonly found in mammalian hosts. Among these, Picobirnaviridae was the most abundant RNA viral family in the horse gut. Beta diversity analyses revealed variations in RNA viral abundance across the three breeds, and differential analysis identified 82 RNA viruses exhibiting significant differences (*p* < 0.05) between Akhal-Teke and Yili horses. Comparisons of coverage scores with other mammals revealed shared viral networks among intestinal RNA viruses of horses, humans, cows, and sheep.

**Discussion:**

This study provides valuable data for future research on the horse gut RNA virome, shedding new light on the cryptic viral sharing network within the horse gut.

## 1 Introduction

RNA viruses are a class of viruses that utilize RNA as their genetic material (1) and are widely distributed in nature (2). RNA viruses are a class of viruses that utilize RNA (single- or double-stranded) as their genetic material and differ significantly from DNA viruses and other biological entities in terms of structure and function (3). Owing to the inherent instability of the RNA structure, RNA viruses are characterized by a high mutation rate and strong adaptability, and ultimately play a key regulatory role in the ecological environment (4). This enables RNA viruses to play critical regulatory roles in the ecological system. RNA viruses rely on either their own RNA-dependent RNA polymerase or the enzymatic systems of host cells for replication (5). Some RNA viruses (such as retroviruses) require reverse transcription of their RNA genomes to be transcribed into DNA before replication (6). Many RNA viruses cause severe infectious diseases and are the primary causative agents of major epidemics (such as seasonal influenza and Coronavirus Disease 2019) (7, 8). Therefore, characterization of the genomic diversity and evolutionary dynamics of RNA viruses has significant implications for human health and ecosystems. Such research not only advances fundamental knowledge in virology and microbial ecology but also provides a theoretical foundation for the development of vaccines, antiviral therapeutics, and evidence-based public health strategies.

With the rapid advancement of high-throughput sequencing technology, the role and diversity of RNA viruses in ecosystems have become major frontiers in virology research, garnering increasing attention (9). Compared to traditional isolation and culture methods, metatranscriptomics enables the direct capture of viral RNA from environmental or biological samples (10), thereby accurately revealing the taxonomic composition and abundance of RNA viruses. This approach overcomes the limitations of conventional virology, which relies heavily on host cell culture, and significantly expands our understanding of RNA viral diversity. For example, Wu et al. conducted an extensive metatranscriptomic analysis of the digestive tissues of the widely farmed oyster Magallana gigas, identifying at least 144 potential novel RNA viruses. Among these, 37 and 25 newly identified RNA viruses were also detected in octopus and seawater, respectively, revealing previously unknown viral sharing networks within the marine food web (11). Similarly, Wang et al. (12) performed large-scale metatranscriptomic sequencing of bats and rodents from Kenya and Uganda, identifying 251 viral genomes associated with vertebrates belonging to 19 viral families. Notably, 87% of these genomes represented potentially novel RNA viruses, some of which were closely related to known human and livestock pathogens. This provides an important foundation for the proactive surveillance of emerging viral pathogens in wild animals.

As one of the most important companion animals for human, horses have long played a pivotal role in various aspects of human life and society. Before mechanization, they served as the most crucial means of transportation and an essential source of labor (13). Today, owing to their exceptional athletic performance, they are still bred worldwide for sports, recreation, and companionship (14). The Akhal-Teke and Thoroughbred breeds are renowned for their exceptional endurance and speed, and they play key roles in modern equestrian sports and breeding programmes worldwide. In contrast, the Yili horse, a dual-purpose breed valued for both meat and riding, is widely raised in China and represents an important indigenous horse population in Xinjiang. Collectively, these breeds represent a diverse spectrum, ranging from those of international economic significance to regionally specific breeds. The close and prolonged human—horse interactions throughout their coevolution underscore the importance of characterizing the RNA virome associated with horse hosts. In this study, we applied metatranscriptomic approaches to characterize RNA viruses present in the gut of three representative horse breeds (Thoroughbred, Akhal-Teke, and Yili), describe the genomic features of horse gut RNA viruses, and analyze their evolutionary patterns. Cross-species comparative analysis of gut viromes from horses, human, cow, and sheep revealed inter-host viral sharing networks, with molecular evidence confirming the presence of common viral circles between human and horse guts. This study enriches our understanding of the complexity of the horse gut virome and establishes a conceptual and methodological paradigm for future research on RNA virus ecology and host interactions.

## 2 Materials and methods

### 2.1 Sample collection

This study collected 16 samples of gut content from three distinct horse breeds (Akhal-Teke, Thoroughbred, and Yili). Thoroughbred and Yili horses were collected in Mu Lei, Xinjiang, China, whereas Akhal-Teke horses were sampled in Urumqi, Xinjiang, China. All horses were maintained under similar husbandry and management conditions and were housed individually in separate stables. Thoroughbred horses were provided ad libitum access to commercial formulated concentrates (either concentrate A: 10% Cool Mix, Connolly’s RED MILLS, Goresbridge, Kilkenny, Ireland; or concentrate B: MDN, Beijing Redrunm Technology Co., Ltd., Beijing, China) together with alfalfa hay. Yili and Akhal-Teke horses were fed mixed pasture forage supplemented with 5 kg/day of concentrate feed (concentrate C: Xinjiang Tiankang Feed Co., Ltd., Xinjiang, China) and alfalfa hay. All animals had free access to water and were supplied with sufficient feed according to local husbandry practices. None of the horses had received antimicrobial treatments (including antibiotics, anthelmintics, or non-steroidal anti-inflammatory drugs) within 2 months prior to sampling. Fresh fecal samples were collected rectally under sterile conditions and immediately transferred into pre-sterilized DNase- and RNase-free 50 mL centrifuge tubes. Samples were transported to the laboratory on dry ice and stored at −80 °C until nucleic acid extraction. Detailed information on the sampled horses and corresponding specimens is provided in Supplementary Table S1.

### 2.2 Fecal RNA extraction and metatranscriptome sequencing

Total RNA was extracted from 0.5 g of fecal content per sample, following the manufacturer’s protocol, using TRIzol reagent (Invitrogen, USA) (15). Briefly, each sample was thoroughly mixed with 200 mg of 0.1 mm zirconia beads (BioSpec Products, USA) and 1.2 mL TRIzol reagent. The mixture was then homogenized using a FastPrep-24 instrument (MP Biomedicals, USA). RNA precipitation was then carried out using a mixture of isopropanol and a salt solution containing 1.2 M sodium chloride and 0.8 M disodium citrate. To remove residual DNA, 1 μL of DNase I (NEB, USA) was added to the precipitated RNA, and the mixture was incubated at 37 °C for 15 min. The integrity and concentration of total RNA were assessed using an Agilent 2100 Bioanalyzer (Agilent Technologies, USA) (16) and NanoDrop 2000 (Thermo Fisher Scientific, USA) (17), respectively. Approximately 100 ng of purified RNA from each sample was used to construct a metatranscriptomic library with the TruSeq RNA Library Preparation Kit v2 (Illumina, USA) (18), following the manufacturer’s instructions. Library quality was evaluated using a high-sensitivity DNA chip on an Agilent Bioanalyzer. Paired-end sequencing (PE150 mode) was performed on the Illumina NovaSeq 6000 platform (Illumina, USA).

### 2.3 Raw data processing

Quality control of all datasets generated from the Illumina sequencing platform was conducted using Fastp (version 0.23.2) (19) with default parameters, producing high-quality reads with a Phred quality score threshold of ≥20 (20). The clean reads were aligned to the horse reference genome (EquCab3.0, accession number GCA_002863925.1, downloaded from the NCBI) using Bowtie2 (version 2.2.4) with the “end-to-end, sensitive, -I 200, -X 400” parameters to remove any potential host contamination reads (21).

### 2.4 Sequence assembly and viral sequence identification

Clean data assembly was performed using MEGAHIT (v1.2.9) with default k-mer (subsequence) parameters (22). Contigs longer than 1,000 nucleotides were retained for further analysis (option: –min-contig-len 1,000) (23). The assembly was generated using the following parameters: –k-min 21, −-k-max 141, −-min-contig-len 1000 (24). The assembled contigs were then screened using Barrnap to identify those encoding rRNA genes, and any contigs containing rRNA sequences were removed (25). Open reading frames (ORFs) were predicted using Prodigal (v2.6.3) with the standard genetic code (parameters: -n -p meta) (26). Next, libraries were constructed for diamond comparison based on published RdRP (RNA-dependent RNA polymerase) sequences (23, 27–29). All predicted open reading frames (ORFs) were screened using DIAMOND BLASTp with the following parameters: coverage ≥ 50%, E-value ≤ 1e-10 and score ≥ 70 (23). These potential RdRP proteins were then compared against an HMM (Hid-den Markov Model) model constructed from these sequences using HMMsearch with the following parameters: E-value ≤ 1e-10 (11). This resulted in candidates that were considered to have nearly complete RdRP sequences. Contigs containing RdRP sequences identified by both DIAMOND and HMMsearch analyses were considered to represent RNA viruses with nearly complete RdRP genes.

### 2.5 Taxonomic and phylogenetic analysis of RNA viruses

Here, we performed taxonomic annotation on 497 RNA virus genomes using VITAP (VMR-MSL37 and NCBI RefSeqs209) (30). We conducted multiple sequence alignment of 515 RdRP-encoding contigs using MUSCLE (v7.520) (31) with the following parameters (−maxiterate 1000 -localpair -thread 1) (32). The resulting alignments were automatically trimmed in the “automated1” mode and further refined using TrimAl (v1.4) (33). An unrooted maximum-likelihood (ML) (34) phylogenetic tree was then constructed using IQ-TREE 2 (35) with the ultrafast bootstrap approximation to estimate the support values for the branches. The optimal models were determined by applying the ModelFinder algorithm (36), which was integrated into the IQ-TREE 2 software (options: -m MFP -B 1000 -bnni). Phylogenetic trees were visualized using Chiplot (37).

### 2.6 Estimation and differential analysis of RNA virus relative abundance

Alpha diversity indices were compared among different horse breeds using the Kruskal–Wallis (38) test to assess statistical significance (39). Based on the Bray–Curtis (40) distance index, similarity matrices were calculated for the different groups, and principal coordinate analysis (PCoA) (41) was employed to analyze *β*-diversity of the viral communities across the three horse breeds (42–44).

To evaluate the prevalence of identified RNA viruses in horses, high-quality reads were mapped to RdRP-containing contigs using Bowtie2 (v2.5.1) in the modified “–very-sensitive-local” mode (parameters: -D 20 -R 3 -N 0 -L 16 -i S,1,0.50 -I 0 -X 2000 –no-unal) (28). The resulting SAM files containing the mapped reads were converted into sorted BAM files using SAMtools (v1.17) for subsequent calculation of genome coverage and estimation of abundance (45). The TPM (Transcripts Per Million) (46) of the viral sequences were calculated using CoverM^1^ with filters set at 90% nucleotide identity and 75% read coverage thresholds (47). TPM metric was used to represent the relative abundance of each viral genome (with the “-m tpm” option enabled in CoverM). Viral abundance matrices were first normalized prior to analysis, and differential comparisons were conducted using the MARS model implemented in DEGseq. In addition, to compare the differential RNA viruses in the gut of horses of different breeds, we analyzed the abundance of RNA viruses in the intestines of Thoroughbred, Akhal-Teke, and Yili horses to identify differences between the two groups (using the non-parametric Mann–Whitney U test) and calculated FDR (48) and log_2_FC. Finally, we identified differential viruses based on thresholds (FDR < 0.05 and |log_2_FC| > 1) (49).

### 2.7 Horse-associated RNA viruses in human, cow and sheep metatranscriptomes

To investigate the potential shared network of RNA viruses between horses, human, and other domesticated animals, we collected a series of gut metatranscriptome datasets from human (60), cow (18), and sheep (6) from the NCBI database (50–52). To ensure the comparability of the data, we screened them using the following criteria. First, the datasets must be metatranscriptomic or RNA virus-related. Second, only gut contents or fecal samples were included. Third, all samples must be collected from healthy individuals (e.g., not from diseased or deceased hosts). A total of 84 datasets were retrieved from public databases.

To assess potential cross-species occurrence of RNA viruses, viral contigs identified from horse metatranscriptomes were used as reference sequences for read mapping against metatranscriptomic datasets from cow, sheep, and human hosts. Mapping was performed using CoverM (mode: covered_fraction) with stringent parameters (≥90% nucleotide identity and ≥75% read alignment length) to reduce false-positive assignments. This strategy follows prior virome analyses employing high-identity thresholds to infer viral presence across host environments. Contigs with mapped reads covering ≥30% of their sequence length were considered putatively present in another host dataset. Shared viral members were then summarized at the family and genus, applying overage fraction cutoffs of 0.3 and 0.9, respectively.

## 3 Results

### 3.1 Sequence quality assessment and statistics for horse gut transcriptome

We collected the gut contents from 16 healthy horses from three different breeds, including Yili horses (5), Akhal-Teke horses (6), and Thoroughbred horses (5). Individual RNA libraries were constructed for each sample and subjected to metatranscriptomic sequencing, yielding a total of 893,459,632 raw reads. After removing low-quality bases and adapter sequences and filtering out host-derived reads, 888,227,632 high-quality clean reads were retained for further analysis. The overall Q30 value reached 94.41%, indicating high sequencing quality (Supplementary Table S1).

### 3.2 Characterization of the horse gut RNA virome

**Figure 1:**
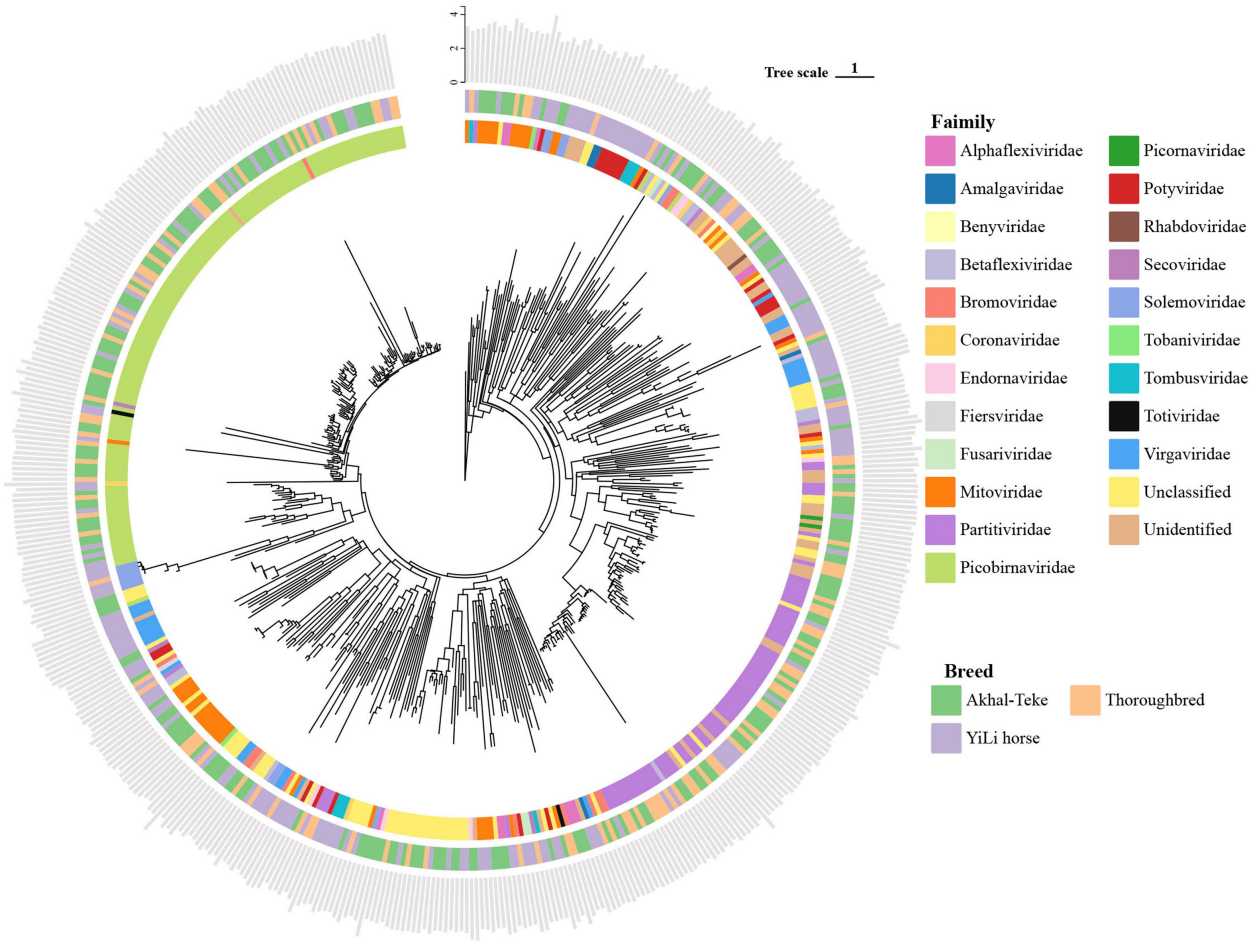
Phylogenetic analysis of RNA viruses based on 515 RdRp proteins. The innermost ring displays the taxonomic classification of each RNA virus at the family level. The outer ring indicates the sample source of each RNA virus. The outermost bar plot shows the length distribution of each RNA virus, presented as log₁₀(length).

High-quality reads were reassembled using MEGAHIT (v1.2.9), and contigs shorter than 1,000 bp were discarded (Supplementary Table S2). Subsequent analyses revealed that a total of 80,765 contigs were assembled from the clean datasets of the 3 horse breeds. The longest contig was 31,464 nucleotides in length. These overlapping clusters were annotated using Prodigal to predict encoded genes. We then identified RdRP proteins using both the BLASTp and HMM-based models (Supplementary Tables S3, S4), yielding 497 contigs carrying RdRP (the intersection of results from both methods). These contigs ranged in length from 1,000 to 29,446 bp, with an average length of 2,123.7 bp (Figure 1). These contigs represent a potential pool of RNA viruses in the horse gut. One giant RNA virus exceeding 20 kb (YL_3_k141_120563) in length was identified among them. This is the largest viral genome (29,446 bp) found in the horse gut to date, belonging to the family Tobaniviridae and the genus Torovirus.

Of the 497 viral contigs recovered from the horse gut metatranscriptomic data, 429 were successfully classified using VITAP. Of these, 381, 232, and 136 contigs were identified at the family, genus, and species levels, respectively. Further taxonomic analysis revealed that the horse gut harbored 136 viruses belonging to 22 families (Supplementary Table S5). These include positive-strand RNA viruses [ssRNA (+)], such as Alphaflexiviridae, Amalgaviridae, Benyviridae, Betaflexiviridae, Bromoviridae, Coronaviridae, Endornaviridae, Fiersviridae, Fusariviridae, Mitoviridae, Phycodnaviridae, Picor-naviridae, Potyviridae, Secoviridae, Solemoviridae, Tobaniviridae, Tombusviridae and Virgaviridae (Figure 1; Supplementary Figure S1).

The Picobirnaviridae family was the most abundant RNA viral family in the horse gut, comprising 134 viral genomes. Notably, RNA viruses belonging to the family Picobirnaviridae were detected in all three horse breeds (Supplementary Table S5). Picobirnaviridae are a class of small, non-enveloped viruses possessing a double-stranded RNA genome consisting of two segments. They are most prevalent in the gut of various vertebrates, including human, and have been widely identified in samples from sources such as algae, human, bats, and monkeys (53). At the genus level, the majority of viruses were classified as Orthopicobirnavirus (115). To further resolve the taxonomic placement of the identified RNA viruses, a comprehensive phylogenetic analysis was conducted based on RdRp protein sequences, incorporating representative reference sequences from established RNA virus families. The majority of viral sequences clustered within recognized RNA virus families, including Picobirnaviridae, Picornaviridae, Partitiviridae, Tombusviridae, and others, enabling family-level taxonomic assignment based on their phylogenetic positions. Viral sequences that did not form stable clades with any known reference groups were classified as unclassified or unidentified RNA viruses, reflecting their high divergence from currently described taxa. At the genus level, a considerable fraction of RdRp sequences could not be assigned to any known viral genera, indicating the presence of a substantial amount of uncharacterized RNA viral diversity. Among the classified taxa, Orthopicobirnavirus was the most abundant genus, followed by Potyvirus and several mitovirus-related genera. At the species level, the dominant taxa were Orthopicobirnavirus beihaiense (71 genomes) and Orthopicobirnavirus hominis (11 genomes).

### 3.3 Heterogeneity of gut RNA viruses among different horse breeds

**Figure 2:**
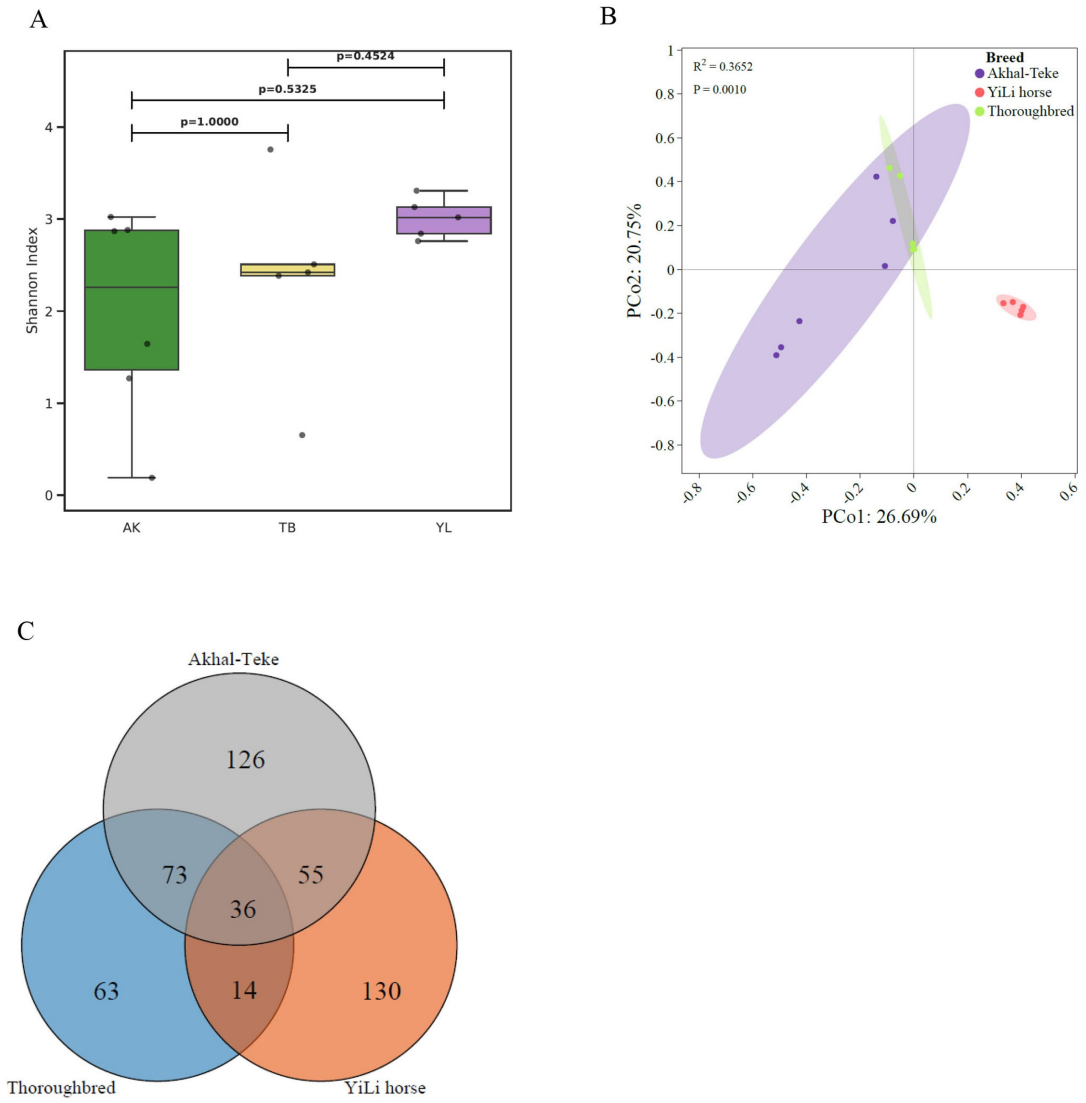
Diversity of equine intestinal RNA viruses. **(A)** Alpha diversity analysis of intestinal RNA viruses from three different horse breeds, based on the Shannon index. **(B)** Beta diversity analysis of intestinal RNA viruses from three different horse breeds, based on the Bray-Curtis distance index. **(C)** Analysis of RNA viruses shared among and unique to the three horse breeds, based on coverage scores.

To further investigate the composition and diversity of intestinal RNA viruses in different horse breeds, we performed a comparative analysis of α-diversity. Although Yili horses exhibited the highest Shannon index and Akhal-Teke horses the lowest, Mann–Whitney U tests revealed no statistically significant differences in α diversity of gut RNA viruses among horse breeds (Figure 2A). Conversely, *β* diversity analysis based on Bray-Curtis distance revealed that the RNA virusesof Thoroughbred and Akhal-Teke horses were significantly more similar to each other than to Yili horses (*p* < 0.01) (Figure 2B). Coverage fraction analysis of the metatranscriptomic data from the 3 breeds showed that 36 core RNA viruses were shared across all samples (Figure 2C), providing an assessment of the heterogeneity of gut RNA viruses among horse breeds. Akhal-Teke, Yili, and Thoroughbred horses possessed 126, 130, and 63 unique viral genomes, respectively. Of the 36 shared core viruses, 24 received taxonomic annotation, of which 22 were classified at the family level. These included 12 viruses in the Picobirnaviridae family, 6 in the Partitiviridae family, 6 in the Mitoviridae family, and one each in the Potyviridae, Solemoviridae, and Virgaviridae families (Figure 2C).

### 3.4 RNA viruses differing in the intestines of different horse breeds

**Figure 3:**
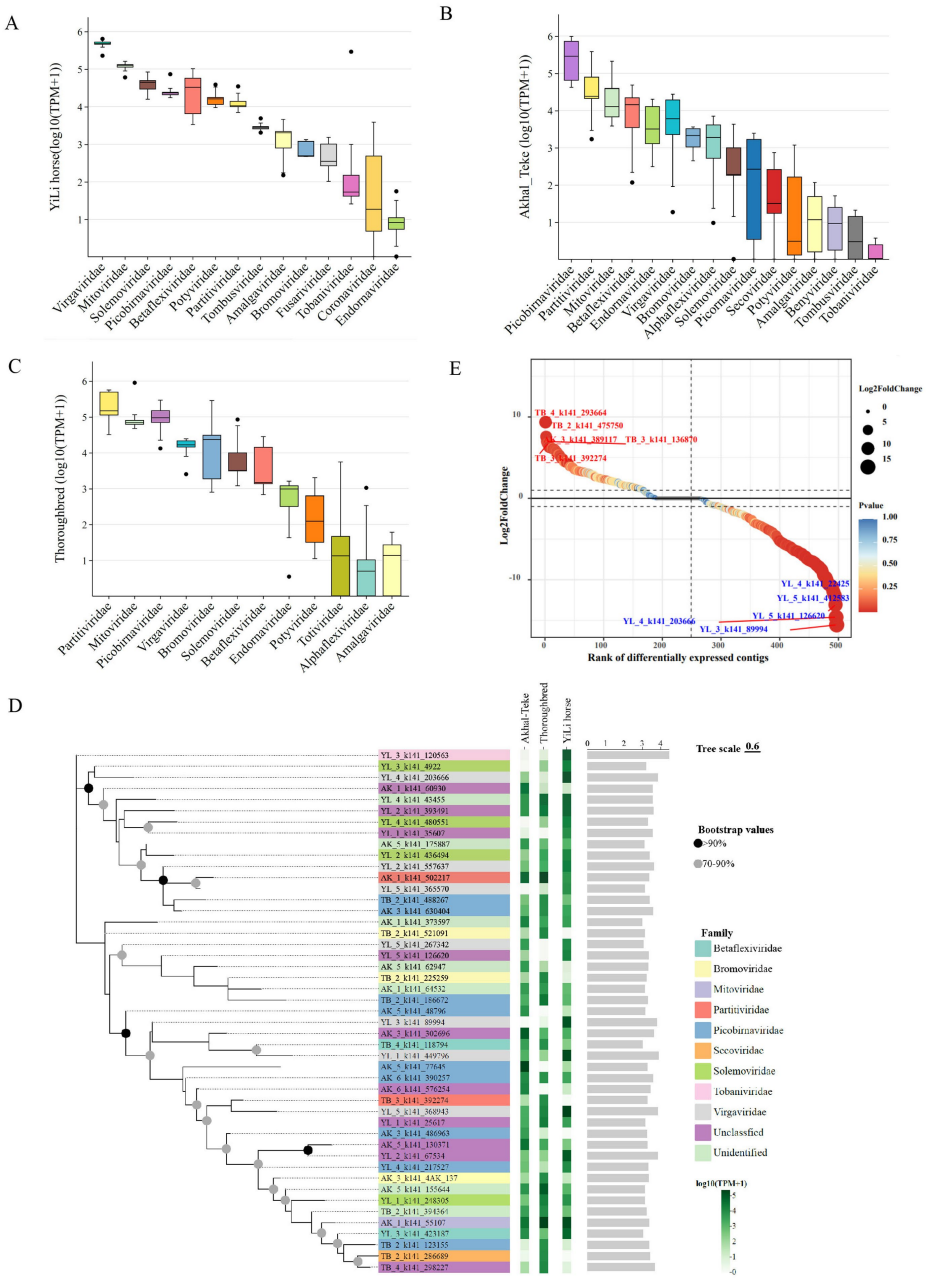
Characteristics of intestinal RNA viruses from different horse breeds. **(A-C)** Abundance of intestinal RNA viruses at the family level in the different horse breeds. **(D)** Phylogenetic tree of the top 20 most abundant intestinal RNA viruses in each horse breed, based on viral contigs. Black and gray dots on the left side of the tree indicate high (>90%) and moderate (70–90%) bootstrap support values, respectively. Colored bars to the right of the terminal branches represent the viral family classification. The gradient green bar heatmap on the right (expressed as log₁₀(TPM+1)) shows the abundance of each virus across the different horse breeds. The bar plot on the far right (expressed as log₁₀(length)) displays the length of each RNA virus contig. **(E)** Differential abundance analysis of RNA viruses between Akhal-Teke and Yili horses. The analysis used a threshold of FDR < 0.05 and |log2FC| > 1. Blue and red labels represent 5 significantly upregulated and downregulated contigs, respectively.

To further analyze and understand the abundance of gut RNA viruses in different horse breeds, we calculated the average TPM values for each sample to assess viral abundance across the three breeds (Supplementary Table S6). At the family level, intestinal RNA viruses exhibited heterogeneity among the three horse breeds. Akhal-Teke, Yili horse, and Thoroughbred horses harbored 16, 13, and 12 viral families, respectively (Figures 3A–C).

The top five families in Akhal-Teke horses were Picobirnaviridae, Partitiviridae, Mitoviridae, Betaflexiviridae, and Endornaviridae. The top five families in Thoroughbred horses were Virgaviridae, Bromoviridae, Mitoviridae, Partitiviridae, and Picobirnaviridae. The top five families in Yili horses were Virgaviridae, Mitoviridae, Solemoviridae, Picobirnaviridae, and Betaflexiviridae. Notably, the Picobirnaviridae and Mitoviridae were dominant families in the gut of all three horse breeds. Furthermore, 11, 12, and 14 RNA viruses in the guts of Akhal-Teke, Thoroughbred, and Yili horses, respectively. It exhibited log-transformed TPM values exceeding the threshold of 4, with detection rates greater than 1% in each breed. Additionally, AK1_k141_55107, belonging to the Mitoviridae family, exhibited an abundance greater than 1% across all 3 breeds, indicating that horses could potentially serve as potential hosts for this virus (Figure 3D).

We analyzed the differences between the three species based on the abundance in-formation of each RNA virus, identifying a total of 82 significantly different viruses in Akhal-Teke and Yili horses only (Supplementary Figure S2). Compared to the Akhal-Teke horse, there are 71 RNA virus genomes upregulated in the gut of the Yili horse, while 11 were found to be downregulated. The top 5 upregulated RNA viruses were TB_4_k141_293664, TB_2_k141_475750, AK3_k141_389117, TB_3_k141_136870, and TB_3_K141392274, while the top five downregulated were YL_4_k141_22425, YL_5_k141_412583, YL_5_k141_126620, YL_4_k141_203666, and YL_3_k141_89994 (Figure 3E; Supplementary Table S7).

### 3.5 Shared network of horse, human, cow, and sheep intestinal RNA viruses

**Figure 4:**
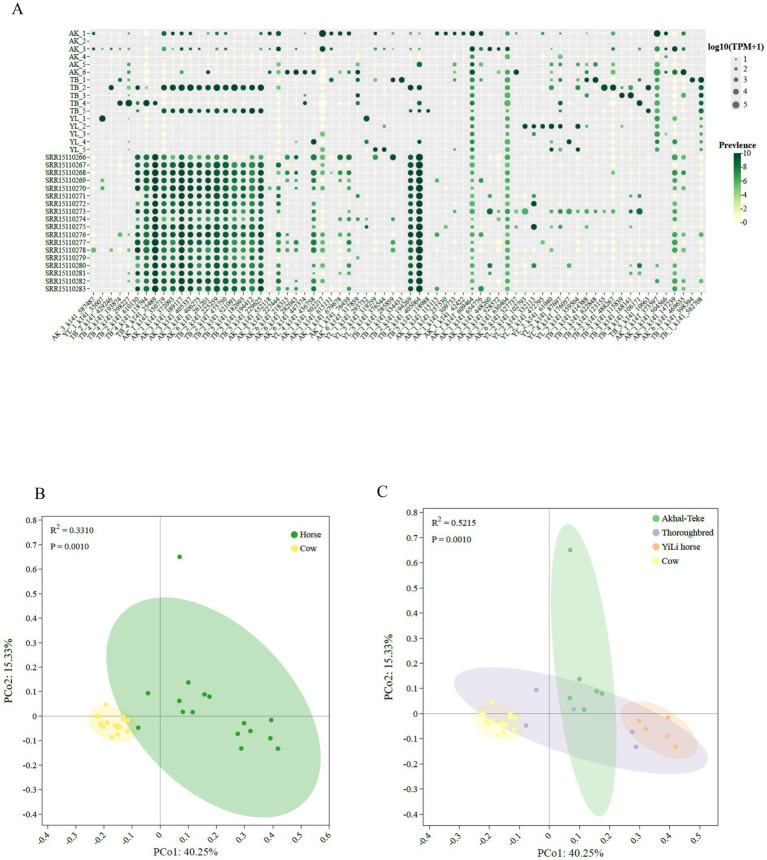
Equine RNA viruses shared with cattle. **(A)** Prevalence and abundance of RNA viruses shared between horses and cattle. **(B)** PCoA analysis of RNA viruses shared between horses and cattle. **(C)** PCoA analysis of RNA viruses shared among the three different horse breeds and cattle.

Using coverage scores from read alignments against public datasets from human, cow, and sheep (Supplementary Table S8), we found that of the 497 viruses identified in the horse guts, 6.23% (31/497), 8.65% (43/497), and 3.82% (19/497) were present in metatranscriptome datasets from human, cow and sheep, respectively (Supplementary Figures S3, S4). In contrast, 49% (19/497) were present in the human and cow datasets (Figure 4A), and 3.82% (19/497) were present in human, cow, and sheep metatranscriptome data (Supplementary Figures S3, S4). Conversely, 49% (317/497) of viruses were specific to horse gut samples. A total of 12.67% (63/497) of viruses were detected in both cow and sheep samples from the same ecological niche (Supplementary Figures S3, S4). PCoA based on abundance data revealed structural similarities between RNA viruses shared by human, cow, and sheep, as well as some horse-derived viruses. Specifically, sheep and Akhal-Teke horses, as well as Thorough-bred horses, human and Yili horses, and cow and Thoroughbred horses, respectively, have more similar viral community structures and closer viral sharing networks (Figures 4B,C; Supplementary Figures S3, S4).

**Figure 5:**
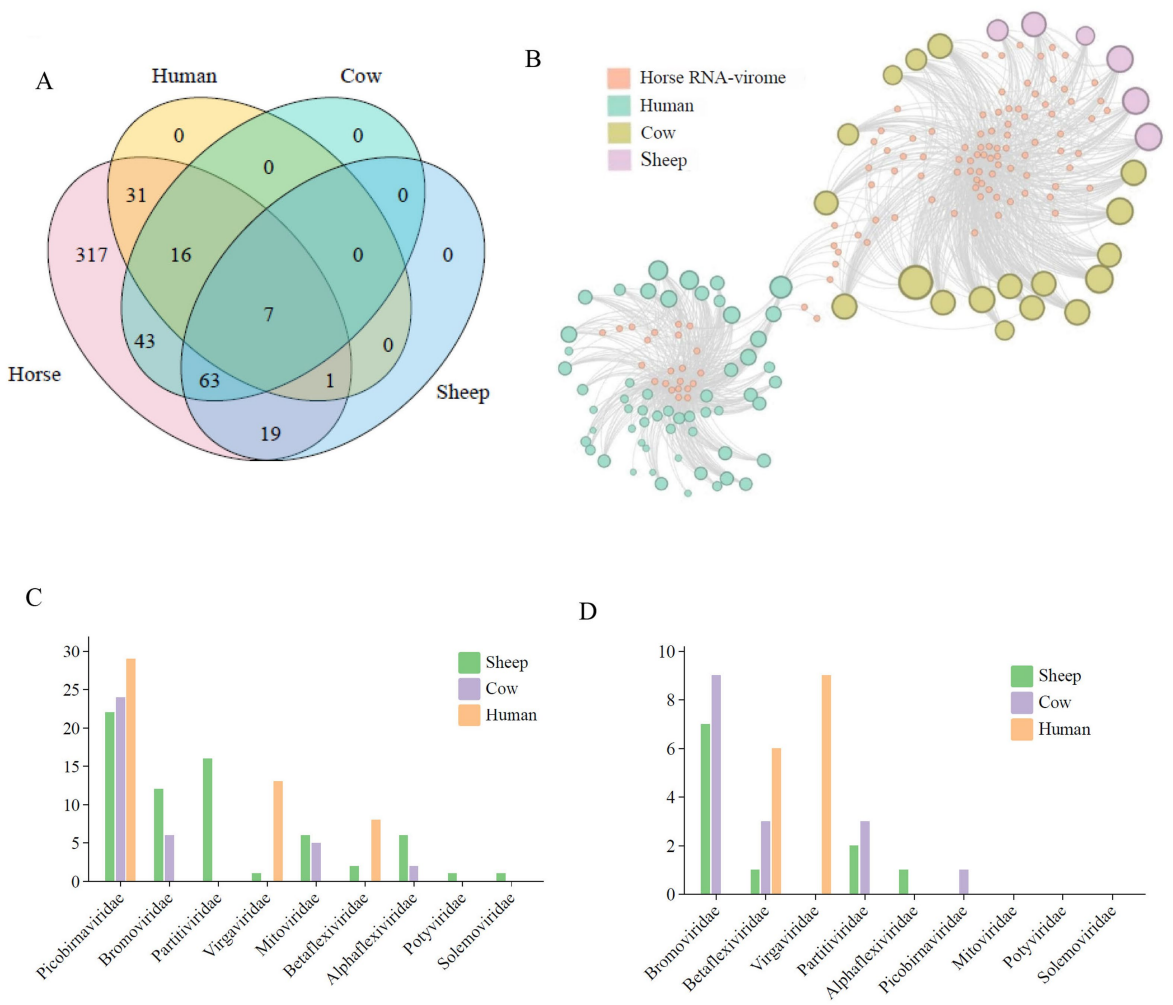
Equine RNA viruses shared with humans and other domestic animals (cattle, sheep). **(A)** Venn diagram showing the distribution of equine viruses in humans, cattle, and sheep. The names of the different groups are indicated at the top of the diagram. **(B)** Network structure of the equine RNA virome. Nodes represent viruses shared with human, cattle, or sheep metatranscriptomes. Edges represent viruses identified in the equine dataset that are also present in subsequent datasets. The size of the nodes corresponds to the number of viruses present in the respective datasets. **(C)** Bar plot showing the presence or dominance of identified equine RNA viruses in other datasets, summarized at the family level. **(D)** Bar plot showing the presence or dominance of identified equine RNA viruses in other datasets, summarized at the genus level.

The average detection rates of horse gut RNA viruses in individual metatranscriptome data from human, cow, and sheep were 0.91 (55/60), 6.83 (123/18), and 15 (90/6), respectively. These results suggest that horse gut RNA viruses are more prevalent in cow and sheep than in human (Figure 5A). Further network analysis revealed that shared horse intestinal RNA viruses exhibited heterogeneous clustering patterns in human versus domestic animals (cow and sheep) (Figure 5B), which may be influenced by their distinct ecological niches within the food chain. By mapping RNA virus genomes to sample sequencing reads, seven viruses shared by horses, human, cow, and sheep were detected, all belonging to the Picobirnaviridae family. Five of these were classified within the Orthopicobirna virus and Orthopicobirna virus beihaiense, while the remaining two were unclassified at the genus and species levels.

We also assessed the detection of these shared viruses at the family and genus taxonomic levels. At the family level, 57, 37, and 67 horse gut RNA viruses were detected in human, cow, and sheep, respectively. At the genus level, no shared RNA viruses were detected in the Solemoviridae or Potyviridae families across any of the three breeds (Figure 5C). At the genus level, the number of shared viruses decreased to 15, 16, and 11 RNA genus in human, cow, and sheep, respectively. None were detected in the Mitoviridae, Solemoviridae, or Potyviridae families. These results suggest that there is a low frequency of shared viruses between human and domesticated animals such as cow and sheep (Figure 5D).

## 4 Discussion

In this study, we analyzed the gut RNA virome in three horse breeds—Akhal-Teke, Yili, and Thoroughbred—using metatranscriptomic sequencing. By applying Diamond BLASTp and HMM-based methods, we identified 497 RNA viruses across the three breeds. Taxonomic annotation revealed a complex and diverse gut RNA virome, comprising 22 viral families and 136 distinct viral species, including viruses harboring dsRNA, positive-sense ssRNA, and negative-sense ssRNA genomes. This highly diverse viral assemblage indicates that the horse gut is a rich ecological niche capable of supporting multiple viral lineages with distinct evolutionary origins.

A total of 36 viruses from five families—Picobirnaviridae, Partitiviridae, Mitoviridae, Betaflexiviridae, and Solemoviridae—were consistently detected in all 3 breeds, suggesting the existence of a stable core virome in the horse gut. This pattern may link these persistent viral families to stable ecological relationships and long-term host adaptation. In particular, Picobirnaviridae and Partitiviridae are known for their broad host ranges or environmental resilience (53, 54), which may explain their prevalence and dominance in this dataset. These core viral families may contribute to the ecological stability of the gut ecosystem and serve as useful targets for future functional studies on RNA virus—microbiota—host interactions (55).

Despite the shared virome, the three horse breeds exhibited significant differences in gut viral composition: the more domesticated Thoroughbred and Akhal-Teke breeds showed distinct gut viromes compared to the Yili horses, likely resulting from a combination of host genetics, diet, and environmental exposure (56). Akhal-Teke horses displayed a higher proportion of Picobirnaviridae. At the same time, Thoroughbreds showed fewer dominant viral taxa but increased relative abundance of certain families such as Partitiviridae, suggesting possible replication advantages or host-specific adaptations. In contrast, the Yili horse virome was predominantly enriched in Virgaviridae (57), a finding consistent with previous studies indicating that host physiology and ecological factors are major drivers of viral community structure in vertebrate gut systems (58).

A considerable proportion of RNA viruses detected in the horse gut remained unclassified or unidentified. These unknown viral taxa may represent novel viral lineages that have not yet been captured by existing reference databases. Their prevalence underscores the need for transcriptomic approaches in discovering novel RNA viruses and highlights the importance of considering the horse gut as a potential reservoir for emerging RNA viruses (9). Investigating these uncharacterized viruses is crucial not only for identifying previously unrecognized viruses but also for understanding virushost interactions of ecological or epidemiological significance (29). We also detected the YL_3_k141_120563 virus, which has a genome length of 29,446 bp. It is the longest known RNA virus genome in the horse gut so far, and it belongs to the genus Torovirus in the family Tobaniviridae (59). This finding highlights the complexity of the horse viral population and matches what is known about Tobaniviridae genome sizes and host ranges (60).

Phylogenetic analysis based on 47 high-abundance viral genomes (logTPM > 4), which consistently exceeded this threshold across all three horse populations, revealed clear clustering of major viral families within the horse gut (Figure 3D). Notably, several low-abundance members of Coronaviridae were also detected across multiple individuals, indicating the potentialcirculation of diverse coronavirus variants in horse populations even in the absence of clinical symptoms (61). These findings emphasize the importance of continuous monitoring of horseviral communities, as RNA viruses may undergo host shifts or acquire pathogenic traits.

Comparative analysis with viral communities in cattle, sheep, and humans revealed significant inter-host viral sharing, with the greatest overlap observed between horses and cattle (129 shared viral taxa), followed by sheep (90) and humans (31). Only seven viruses were universally shared. These results suggest that host specificity is a major determinant of viral community composition. Multiple viral families, including Bromoviridae and Picobirnaviridae, were consistently present in both horse and bovine datasets. Coverage threshold analysis indicated that shared viral taxa were predominantly low-abundance members, whereas high-abundance viruses tended to be host-specific. This supports the notion of a stable, host-specific core RNA virome coexisting with transient viral members. Although no disease symptoms were reported in the sampled animals, the detection of shared RNA viruses among horses, livestock, and humans underscores the need for integrated One Health surveillance in regions with frequent cross-species contact.

## 5 Conclusion

A systematic comparative analysis of horse gut metatranscriptomes revealed a previously underexplored RNA viral diversity across three horse breeds. Metatranscriptome data from our study in Xinjiang, China, may suggest that the horse gut virome could be part of complex multi-species viral-sharing networks with humans, cows, and sheep, providing one possible line of evidence for such potential cross-species viral connectivity. The viruses identified in horses may serve as useful indicators for monitoring cross-species transmission of RNA viruses circulating within shared ecological niches. Overall, this study broadens current understanding of the horse intestinal RNA virome and provides new insights into viral diversity, host specificity, and interspecies viral connectivity. Future work should investigate the functional roles of core RNA viruses, the ecological drivers underlying breed-specific viral patterns, and the potential zoonotic risks associated with the unclassified viral clades identified in this study.

## Data Availability

The raw data of RNA virome presented in this study were deposited in NCBI SRA database under the accession numbers PRJNA1345254 and PRJNA973826, and they can be accessed by https://www.ncbi.nlm.nih.gov/bioproject/PRJNA1345254 and https://www.ncbi.nlm.nih.gov/bioproject/PRJNA973826.

## Footnotes

1
https://github.com/wwood/CoverM
