# Supplementary figures and images for: Metatranscriptomics reveals the horse gut RNA virome and a viral sharing network with human and domestic animals
Source: Front Vet Sci. 2026 Mar 3;13:1755551. doi: 10.3389/fvets.2026.1755551 (PMC12991997; doi:10.3389/fvets.2026.1755551)

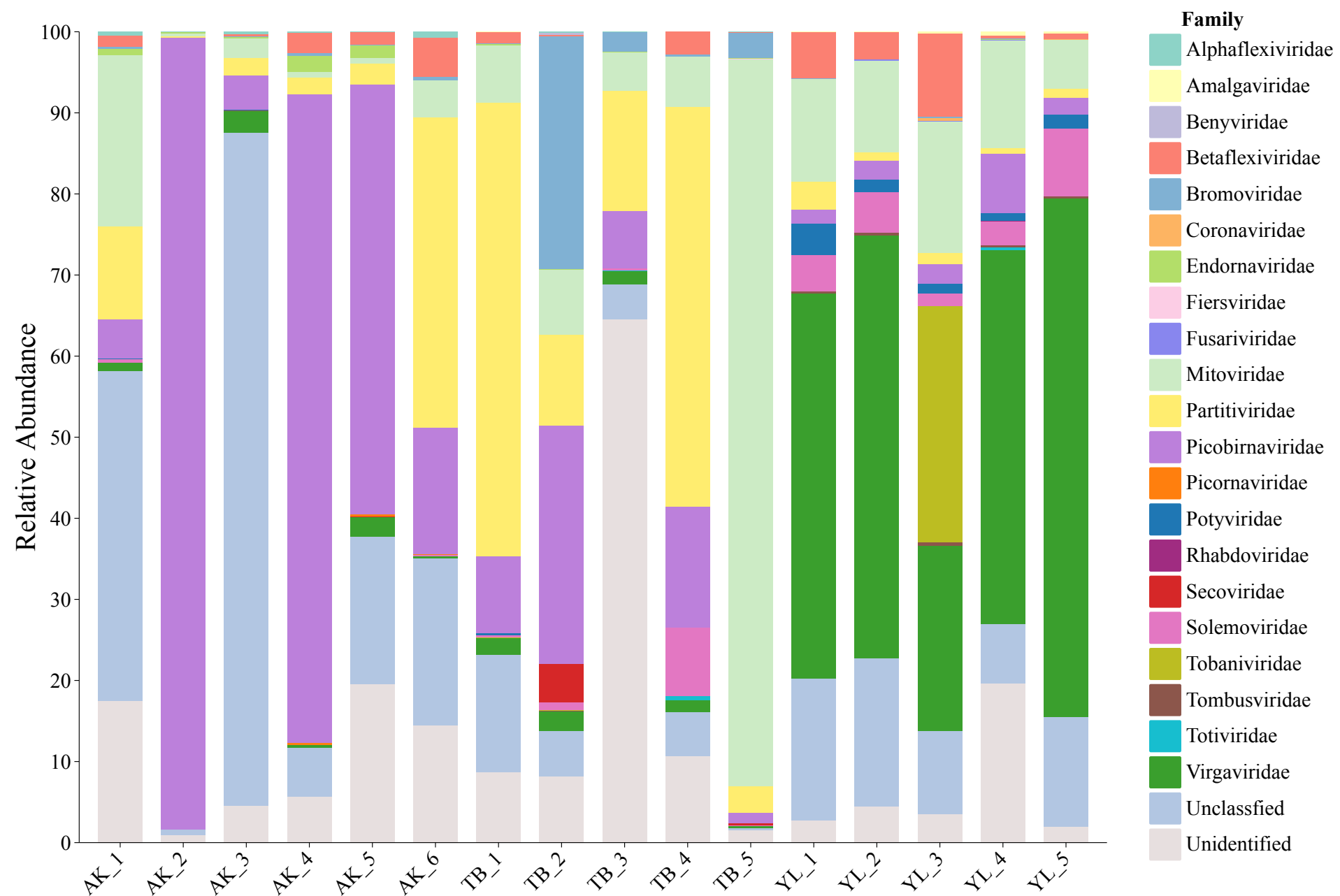

Figure S1 The relative abundance of RNA viruses from 16 samples in family.

Supplement: Supplementary file 1 [file Data_Sheet_1.pdf]

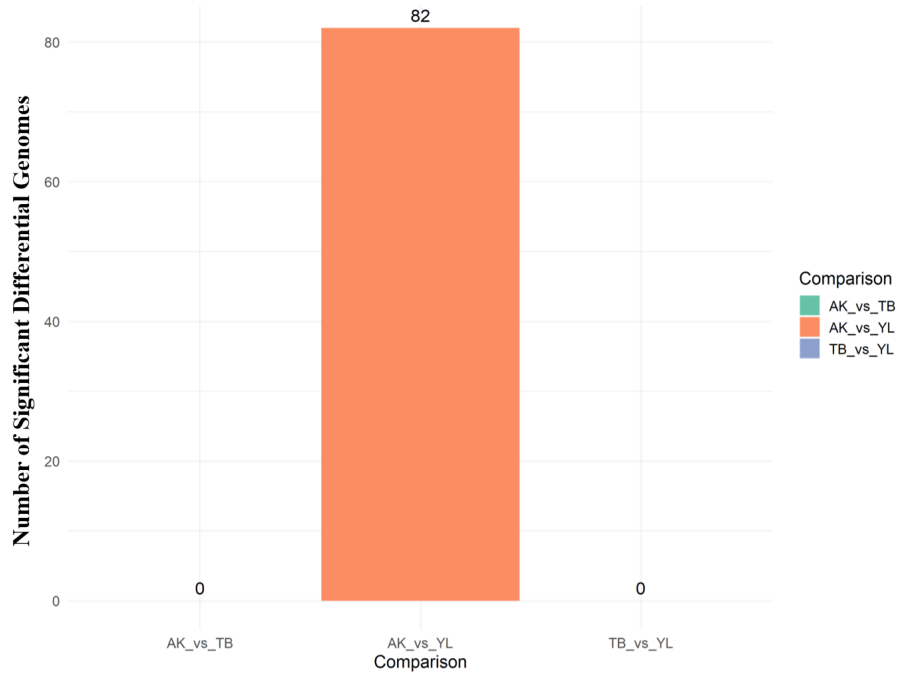

Figure S2 The abundance differential contigs between 3 breeds horse.

Supplement: Supplementary file 2 [file Data_Sheet_2.pdf]
