# Supplementary material for: Metatranscriptomics reveals the horse gut RNA virome and a viral sharing network with human and domestic animals
Source: Front Vet Sci. 2026 Mar 3;13:1755551. doi: 10.3389/fvets.2026.1755551 (PMC12991997; doi:10.3389/fvets.2026.1755551)
Supplement: Supplementary file 3 [file Data_Sheet_3.pdf]

Figure S3

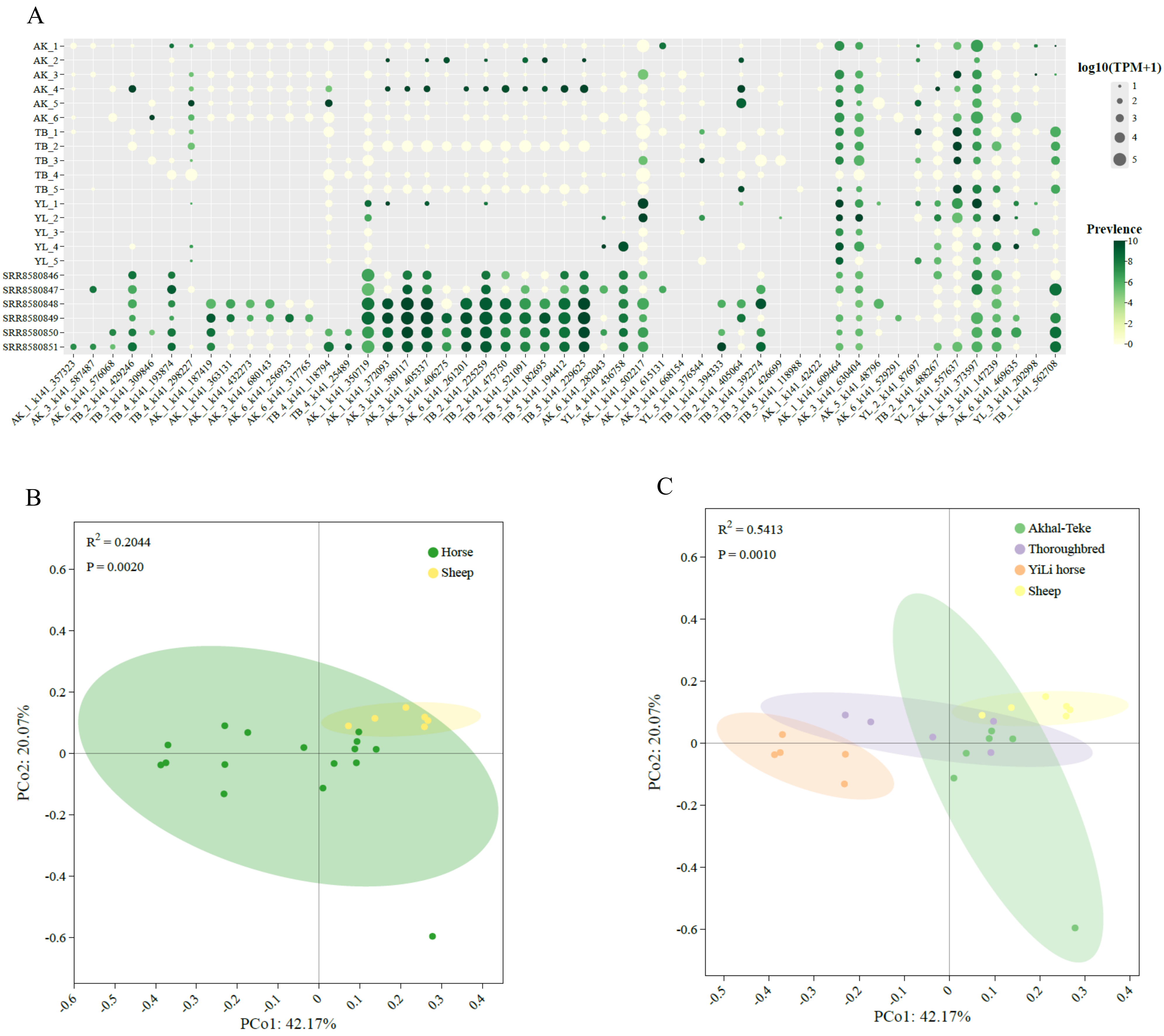

Figure S3 The horse RNA virus shared with sheep. A The prevlence and abundance of horse RNA virus shared with sheep. B The PcoA analysis of shared RNA virus between horse and sheep. C The PcoA analysis of shared RNA virus between 3 different breeds horse and sheep.
